# Supplementary material for: Synergy between Auranofin and Celecoxib against Colon Cancer In Vitro and In Vivo through a Novel Redox-Mediated Mechanism
Source: Cancers (Basel). 2019 Jul 3;11(7):931. doi: 10.3390/cancers11070931 (PMC6678510; doi:10.3390/cancers11070931)
Supplement: Supplementary file 1 [file cancers-11-00931-s001.zip › Cancers-510549-Suppl Info.pdf]

Supplementary Materials

Synergy between Auranofin and Celecoxib against Colon Cancer In Vitro and In Vivo through a Novel Redox-Mediated Mechanism

Yi Han, Ping Chen, Yanyu Zhang, Wenhua Lu, Wenwen Ding, Yao Luo, Shijun Wen, Ruihua Xu, Panpan Liu and Peng Huang

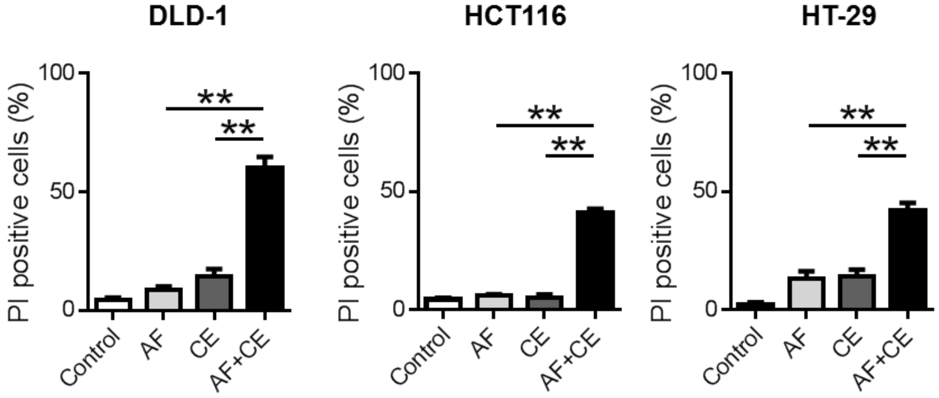

Figure S1. Quantitative comparison of cell death induced by AF 1 μM, CE 10 μM or AF 1 μM + CE 10 μM for 48 h (based on Figure 2C). ** Indicates a statistically significant difference at $p < 0.01$.

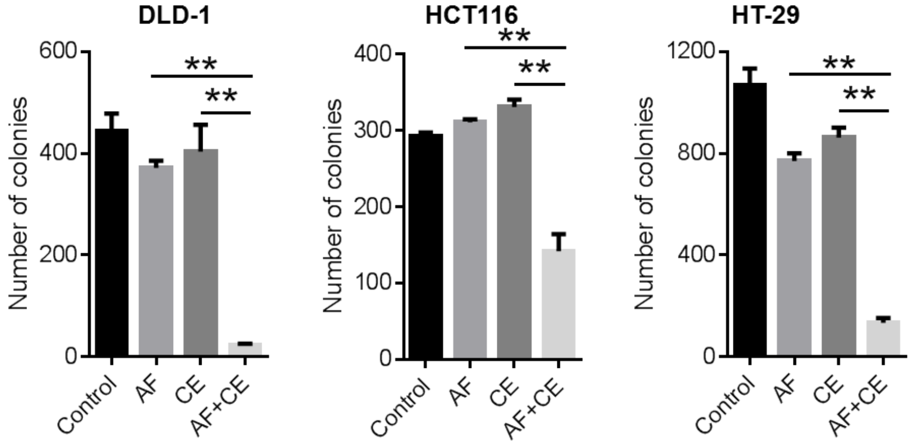

Figure S2. Quantitative comparison of colony formation in indicated groups (based on Figure 2D). ** Indicates a statistically significant difference at $p < 0.01$.

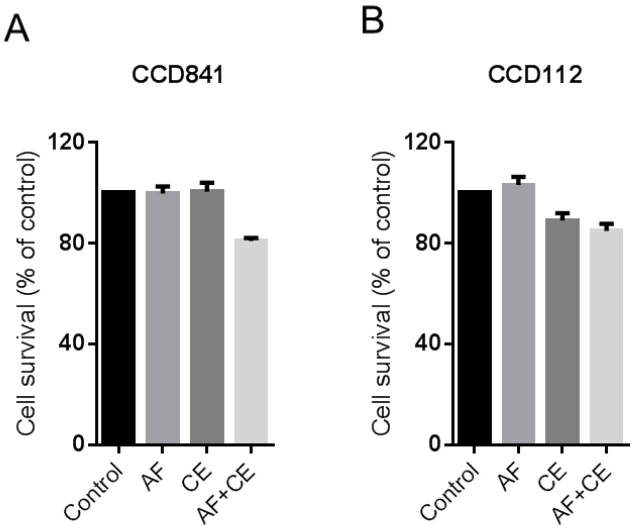

Figure S3. AF/CE combination has minimum cytotoxic effect towards non-cancerous cells. CCD841 and CCD112 cells were treated with AF 1 μM, CE 10 μM or AF 1 μM + CE 10 μM for 72 h. Cell viability was measured using the MTS assay.

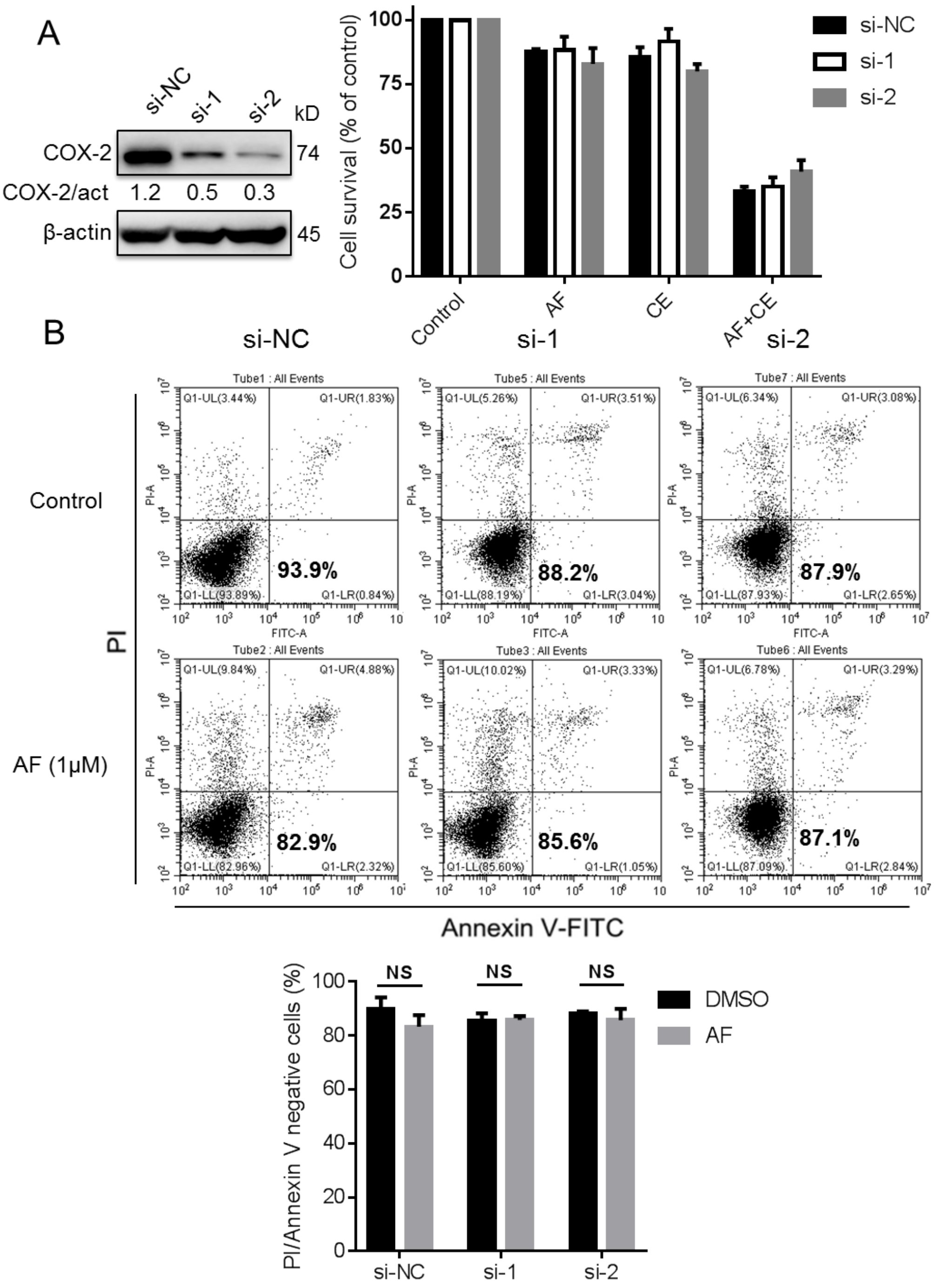

Figure S4. The anticancer effect of auranofin (AF) and celecoxib (CE) combination was independent of COX-2 expression. (**A**) MTS assay on DLD-1 cells transfected with COX-2-specific siRNA or control siRNA for 48 h followed by treatment with AF 1 μM, CE 10 μM or AF 1 μM + CE 10 μM for 72 h. The immunoblot shows siRNA-mediated COX-2 knockdown. (**B**) Apoptosis assay on DLD-1 cells transfected with COX-2-specific siRNA or control siRNA for 48 h followed by treatments with 1 μM AF for 48 h. NS means no significant difference.

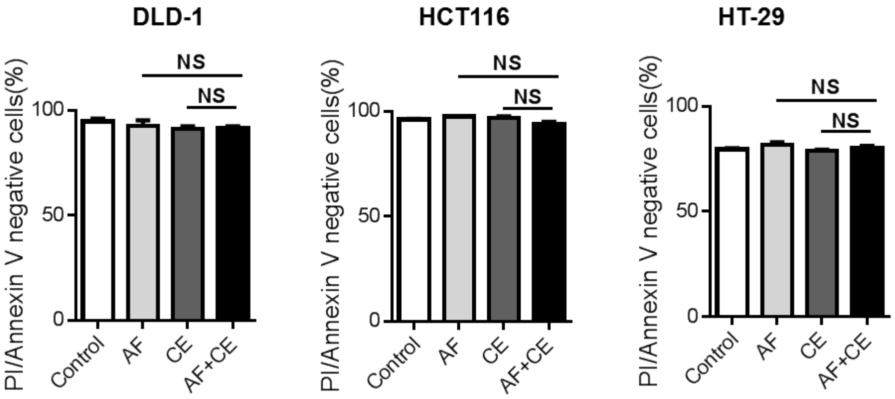

Figure S5. Quantitative comparison of cell death induced by AF 1 μM, CE 10 μM or AF 1 μM + CE 10 μM for 24 h (based on Figure 3G). NS means no significant difference.

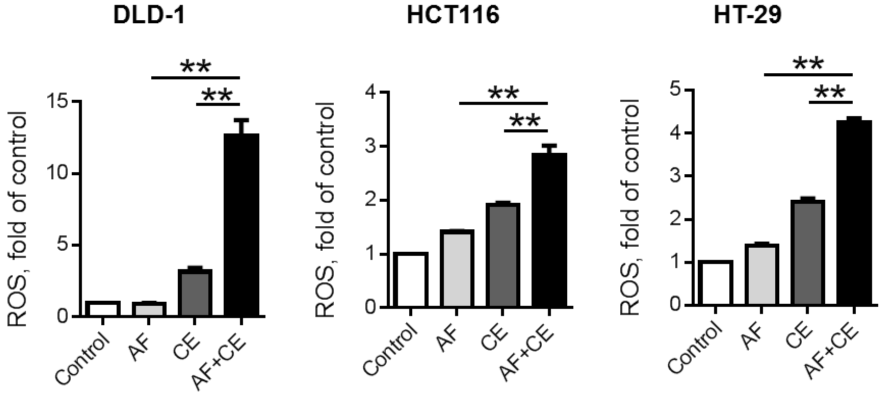

Figure S6. Quantitative comparison of ROS level induced by AF 1 μM, CE 10 μM or AF 1 μM + CE 10 μM for 12 h (based on Figure 5C). NS means no significant difference. ** Indicates a statistically significant difference at $p < 0.01$.

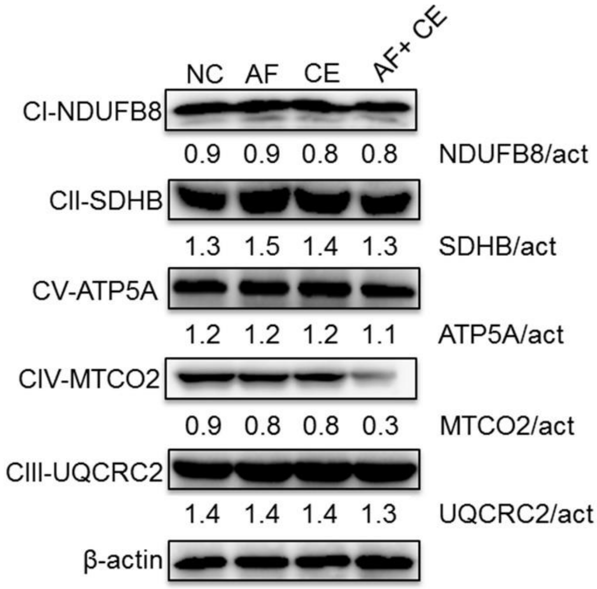

Figure S7. DLD-1 cells were treated with indicated conditions for 24 h, and cell extracts were analyzed by western blot for NDUFB8, SDHB, ATP5A, MTCO2 and UQCRC2 protein. Actin was used as loading control. A representative experiment out of three is shown and the densitometric ratios of specific proteins/actin are indicated below each blot.
