# Supplementary material for: Synergy between Auranofin and Celecoxib against Colon Cancer In Vitro and In Vivo through a Novel Redox-Mediated Mechanism
Source: Cancers (Basel). 2019 Jul 3;11(7):931. doi: 10.3390/cancers11070931 (PMC6678510; doi:10.3390/cancers11070931)

Fig. 5B

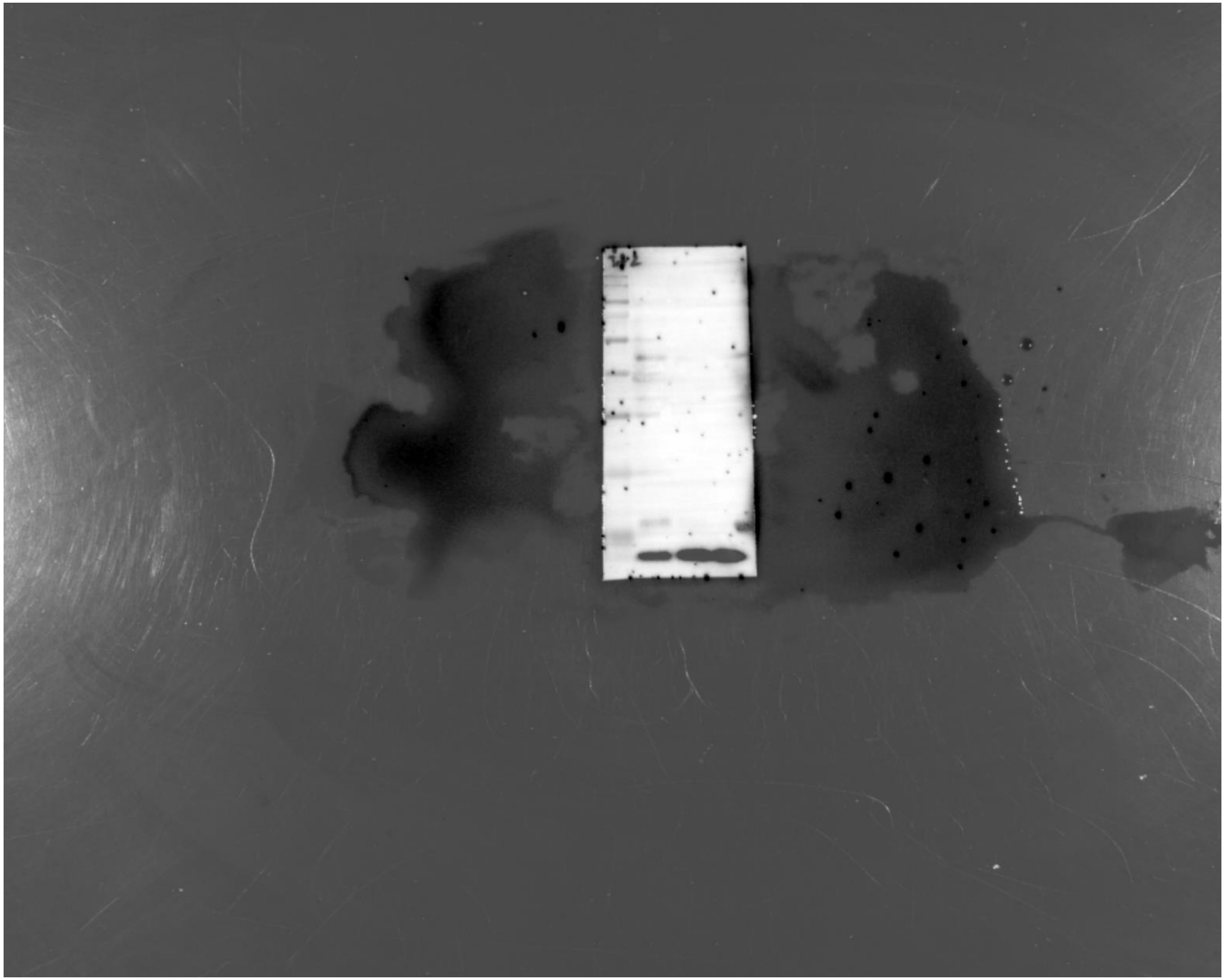

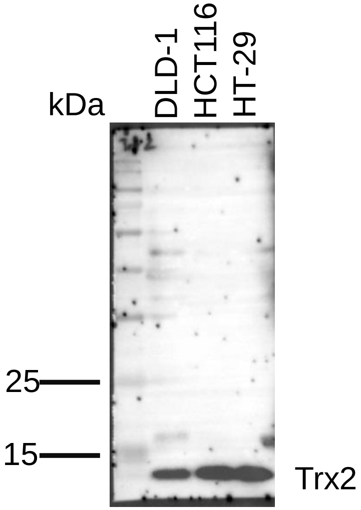

According to the redox western blot protocol, we can't load protein molecular weight marker onto gel to avoid the effect of reducing agent (β-mercaptoethanol or DTT) contained in the molecular weight marker solution. But we perform a test to confirm the Trx2 antibody is correct.

Fig. 5B

DLD-1

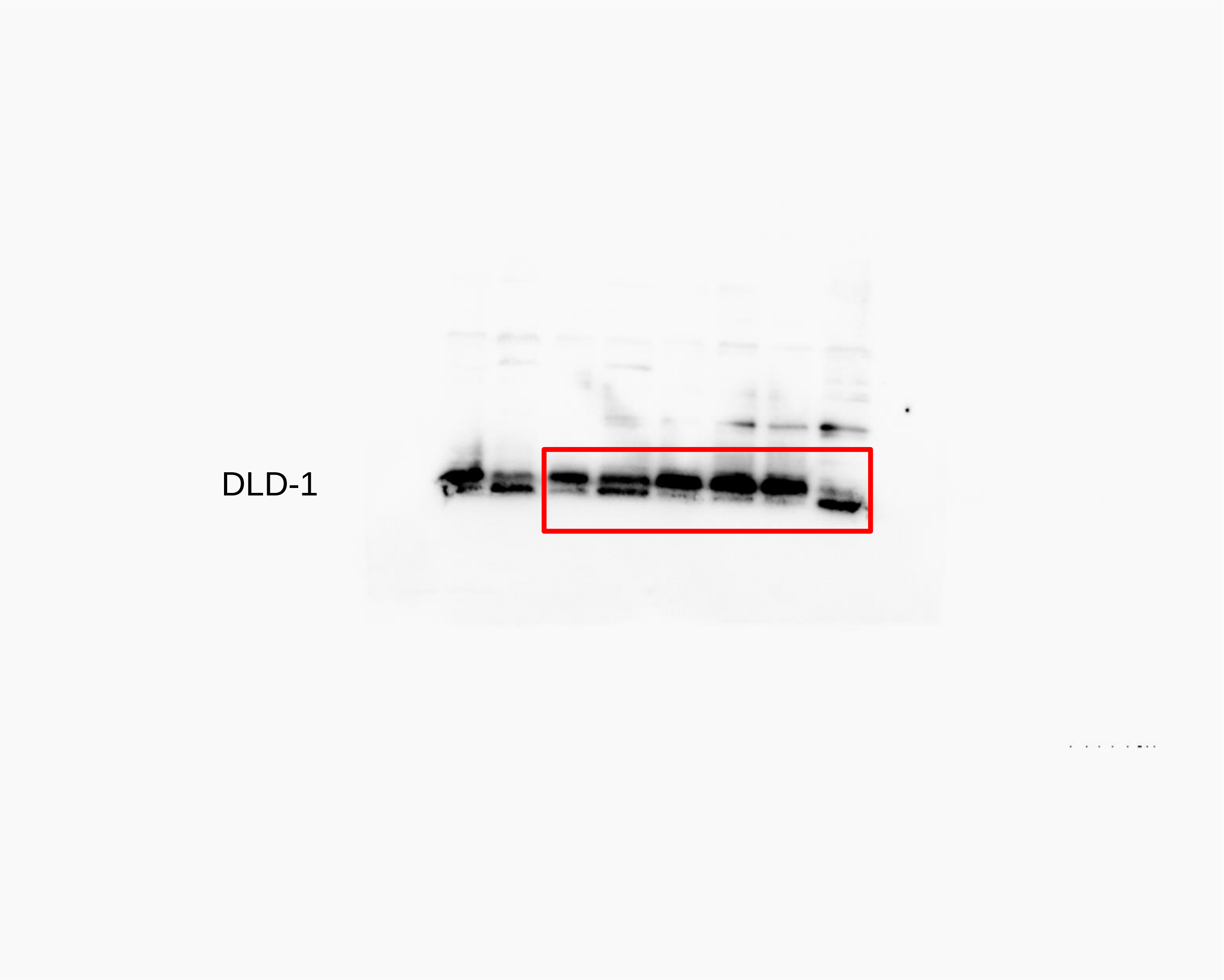

Fig. 5B

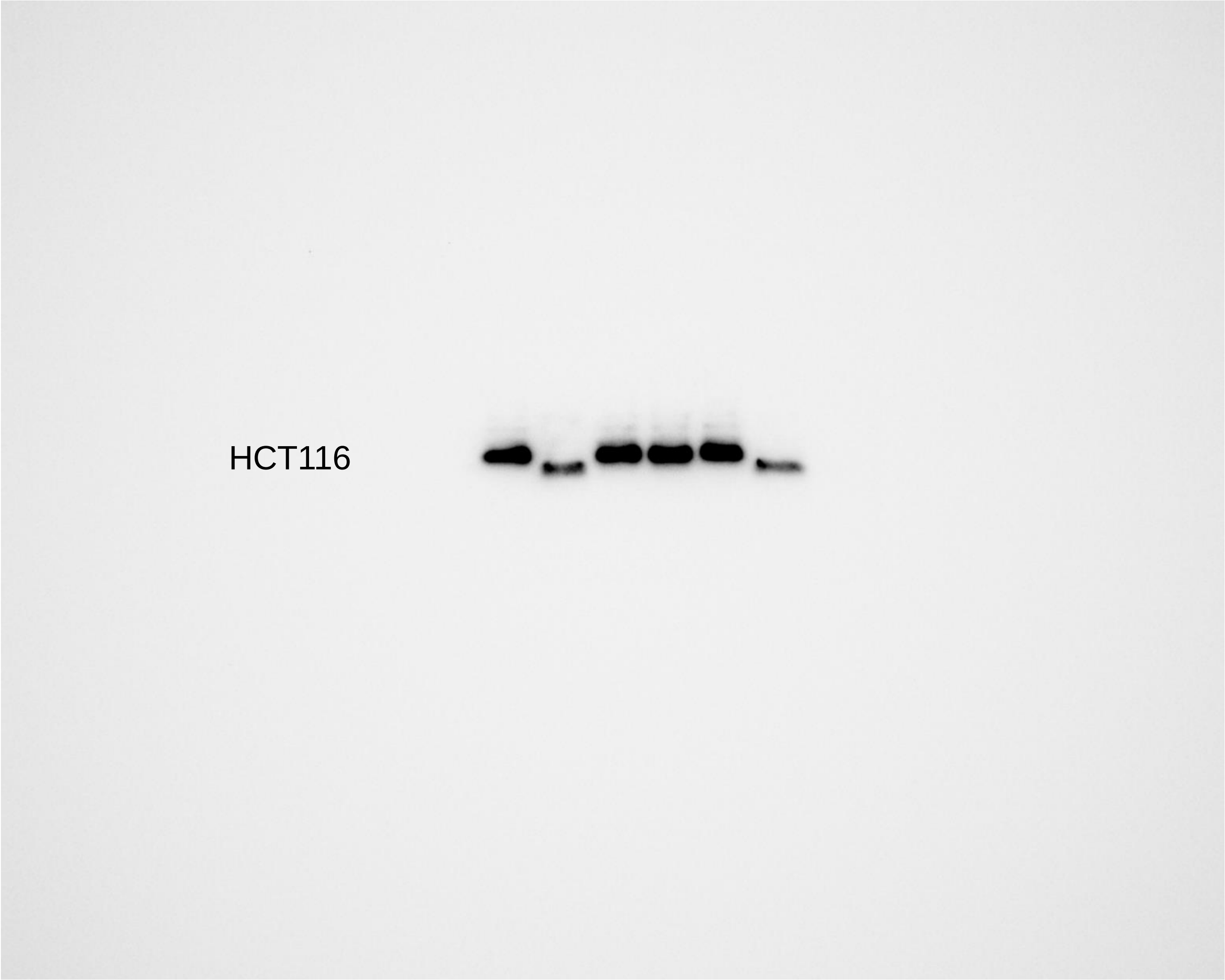

Fig. 5B

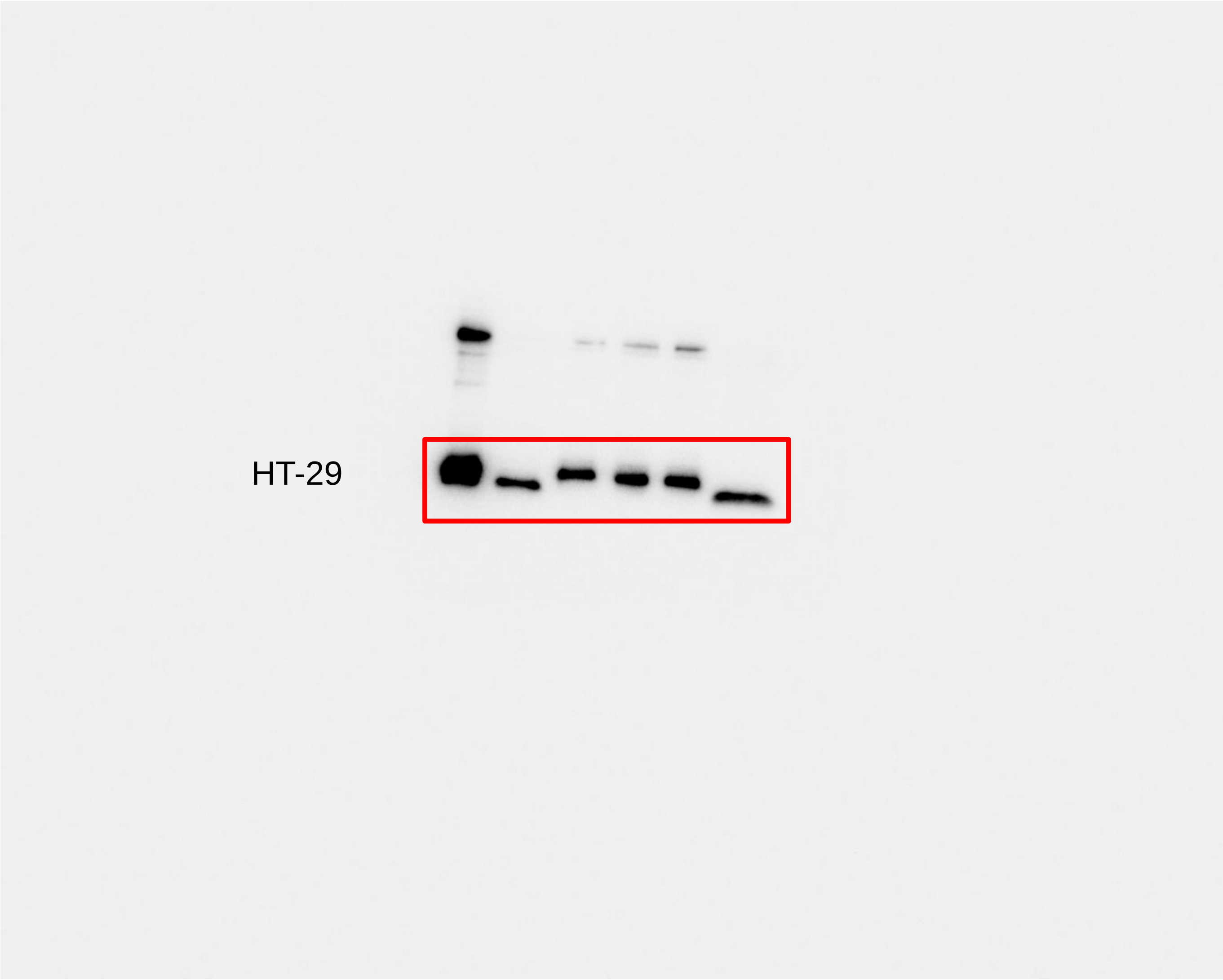

Fig. 5D

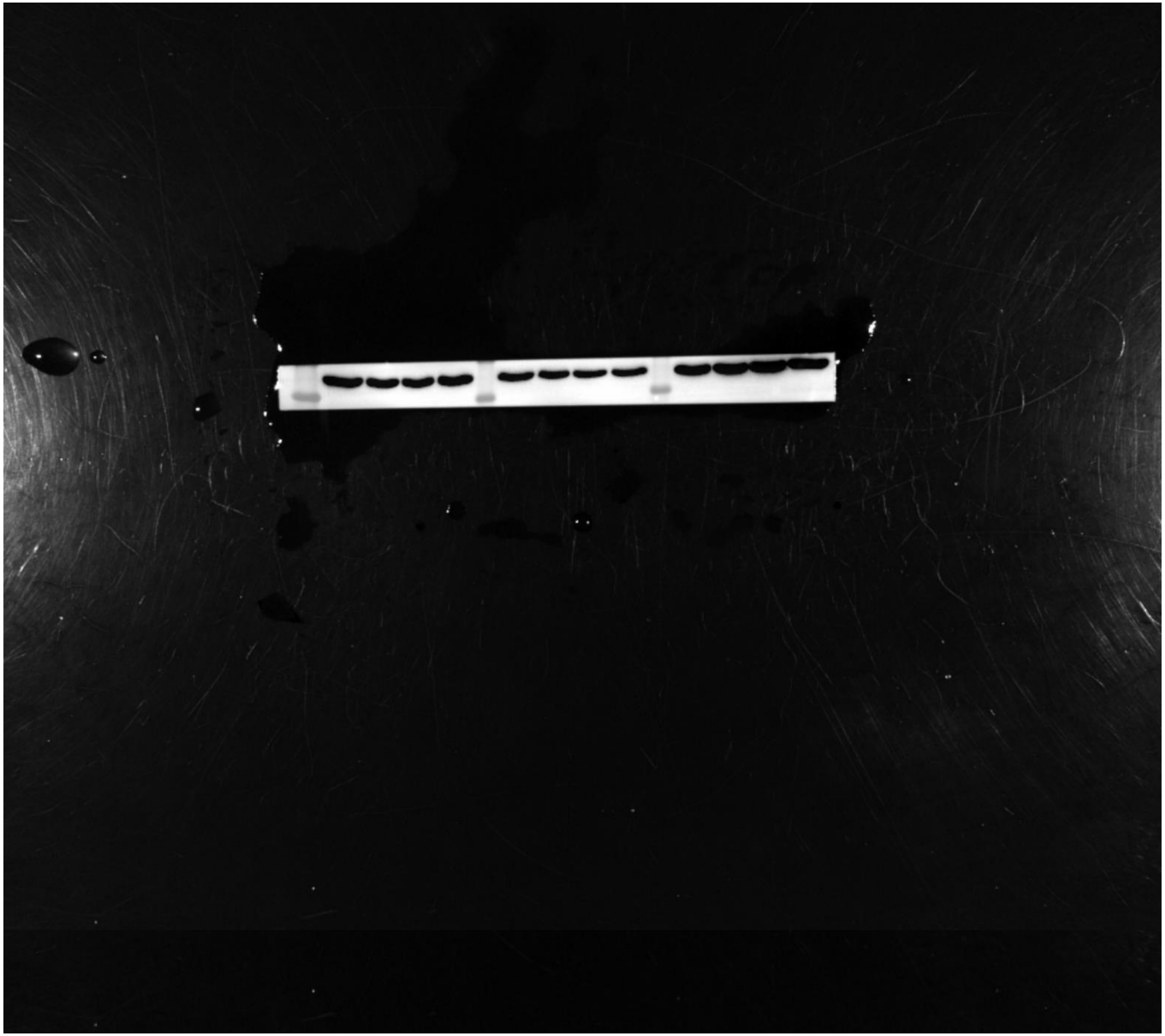

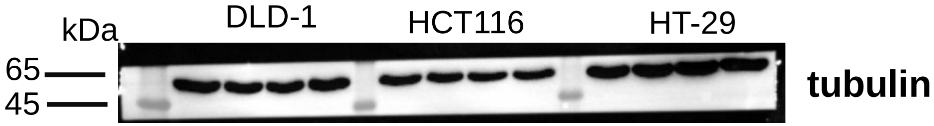

Fig. 5D

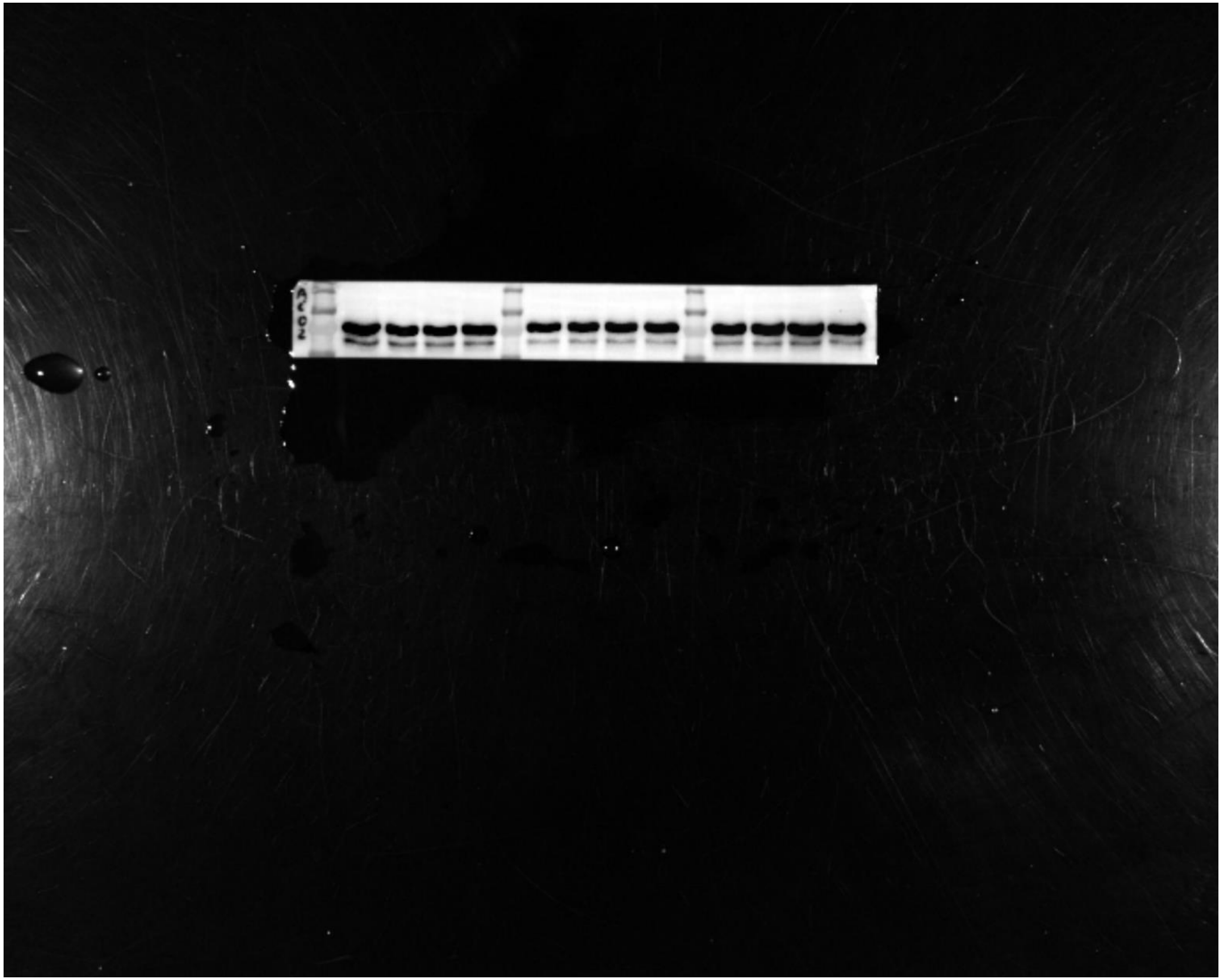

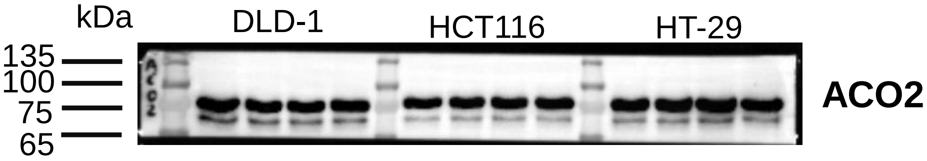

Fig. 5E

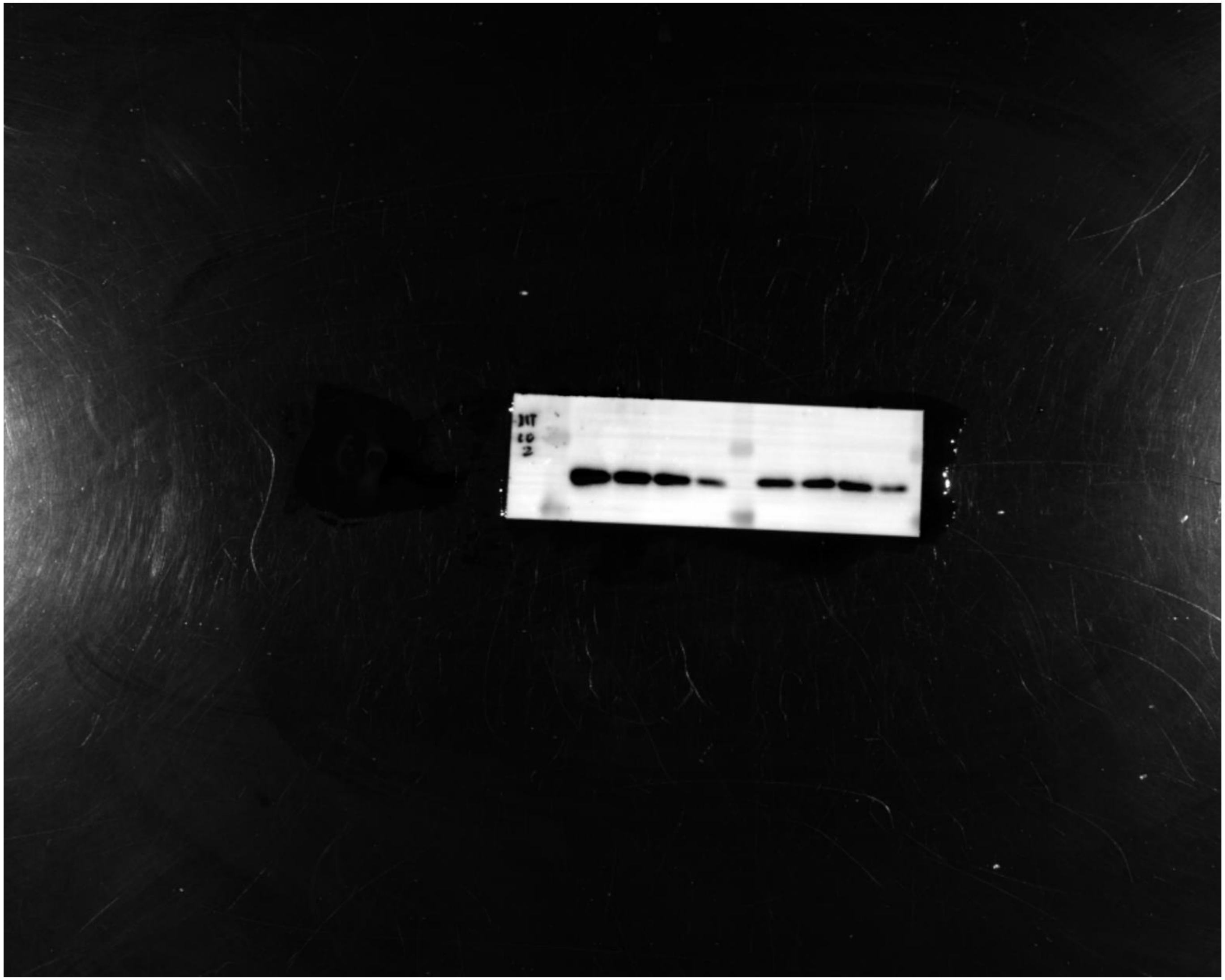

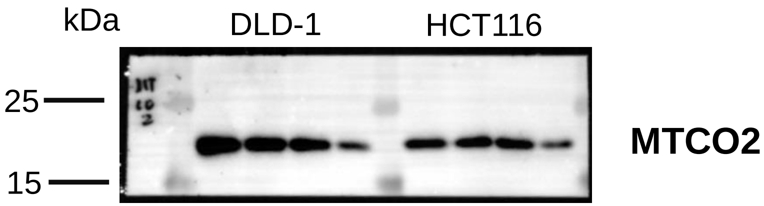

Fig. 5E

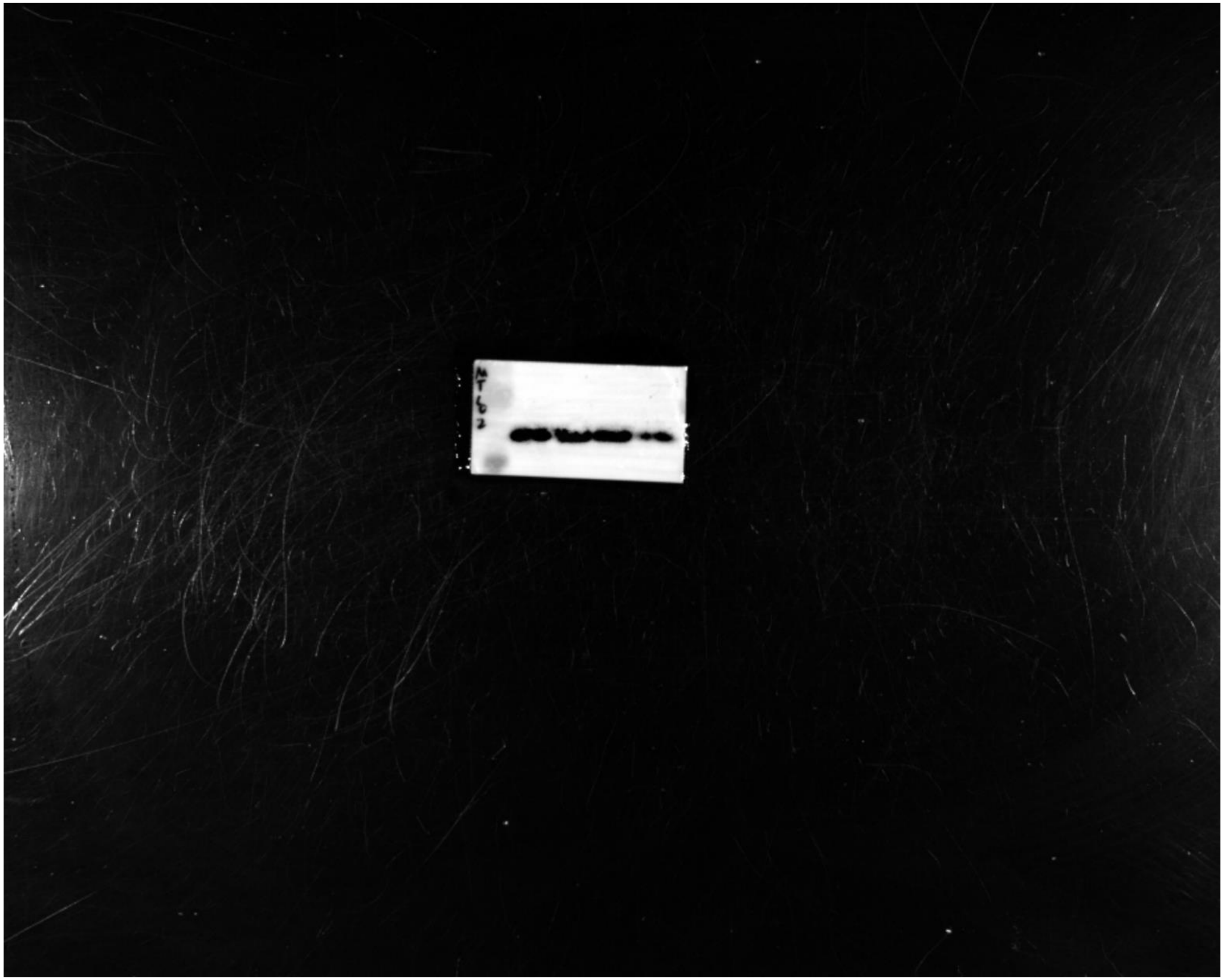

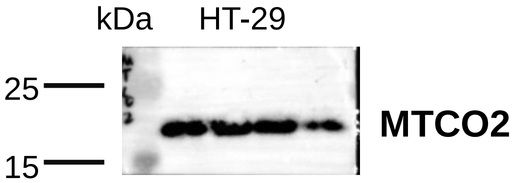

Fig. 5E

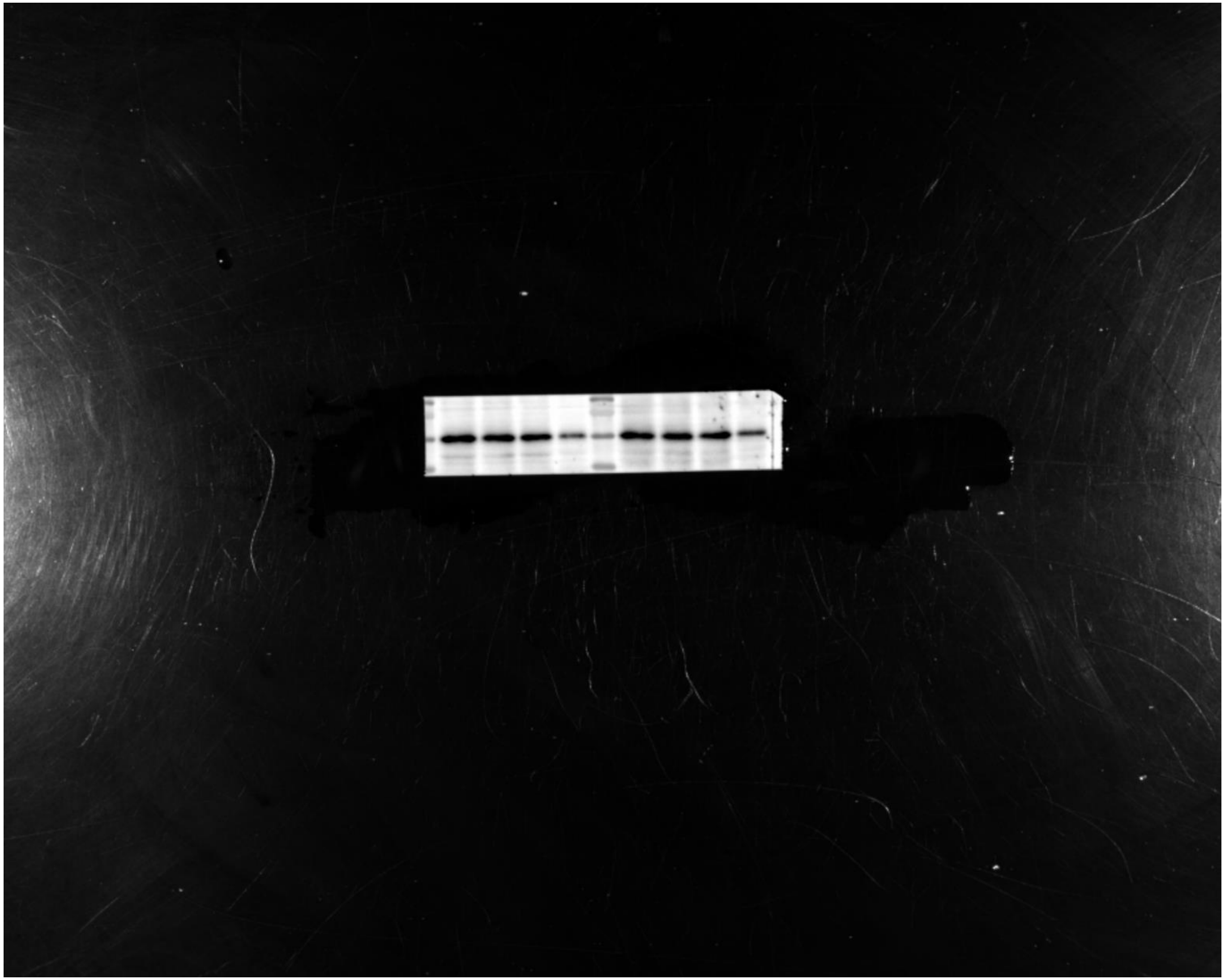

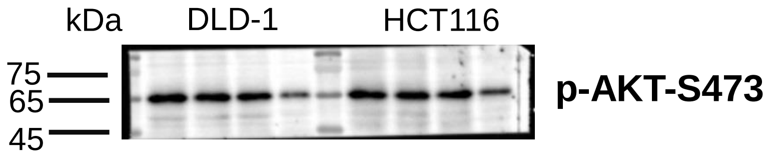

Fig. 5E

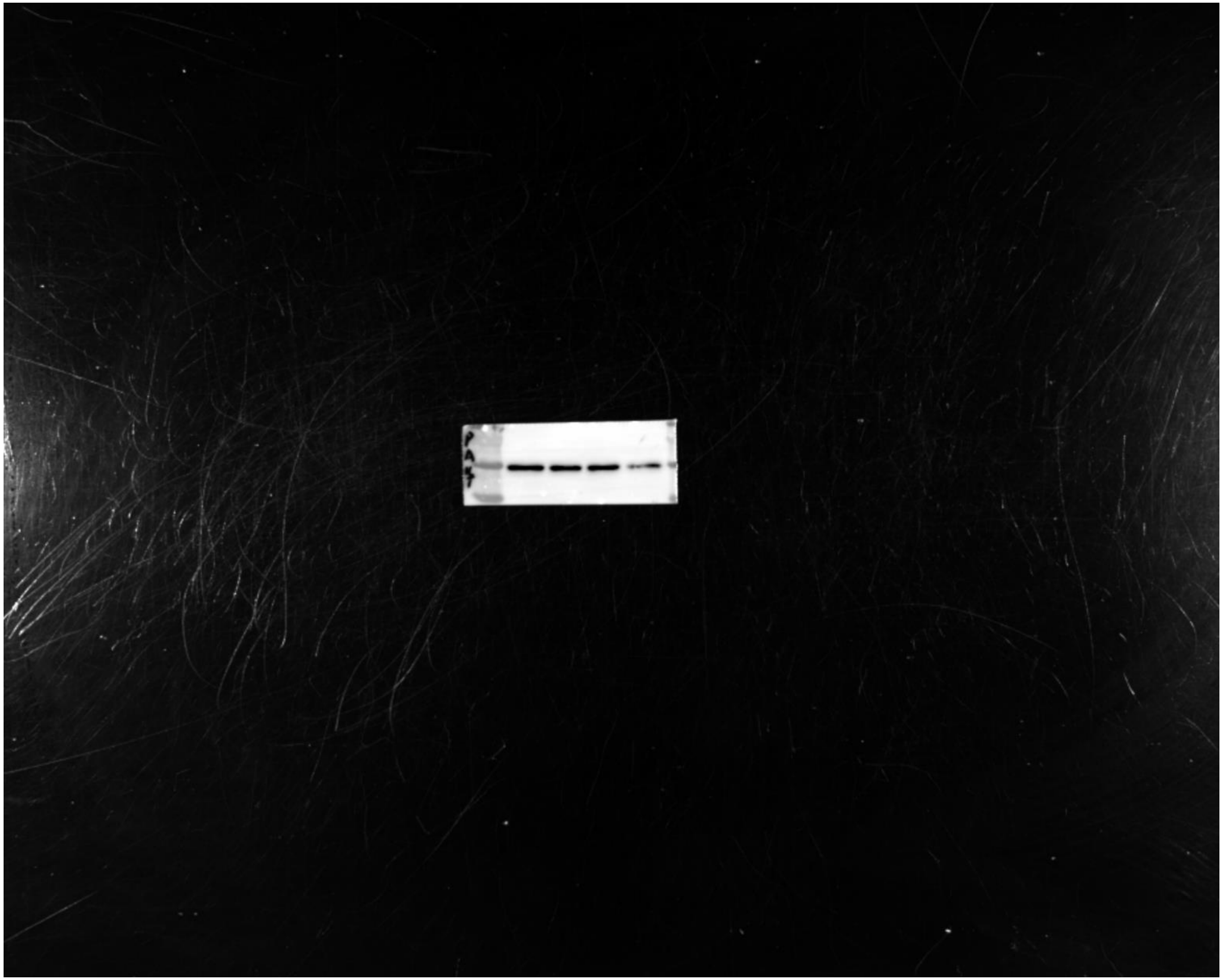

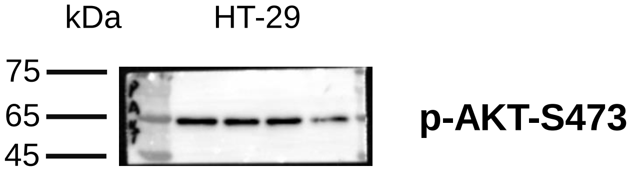

Fig. 5E

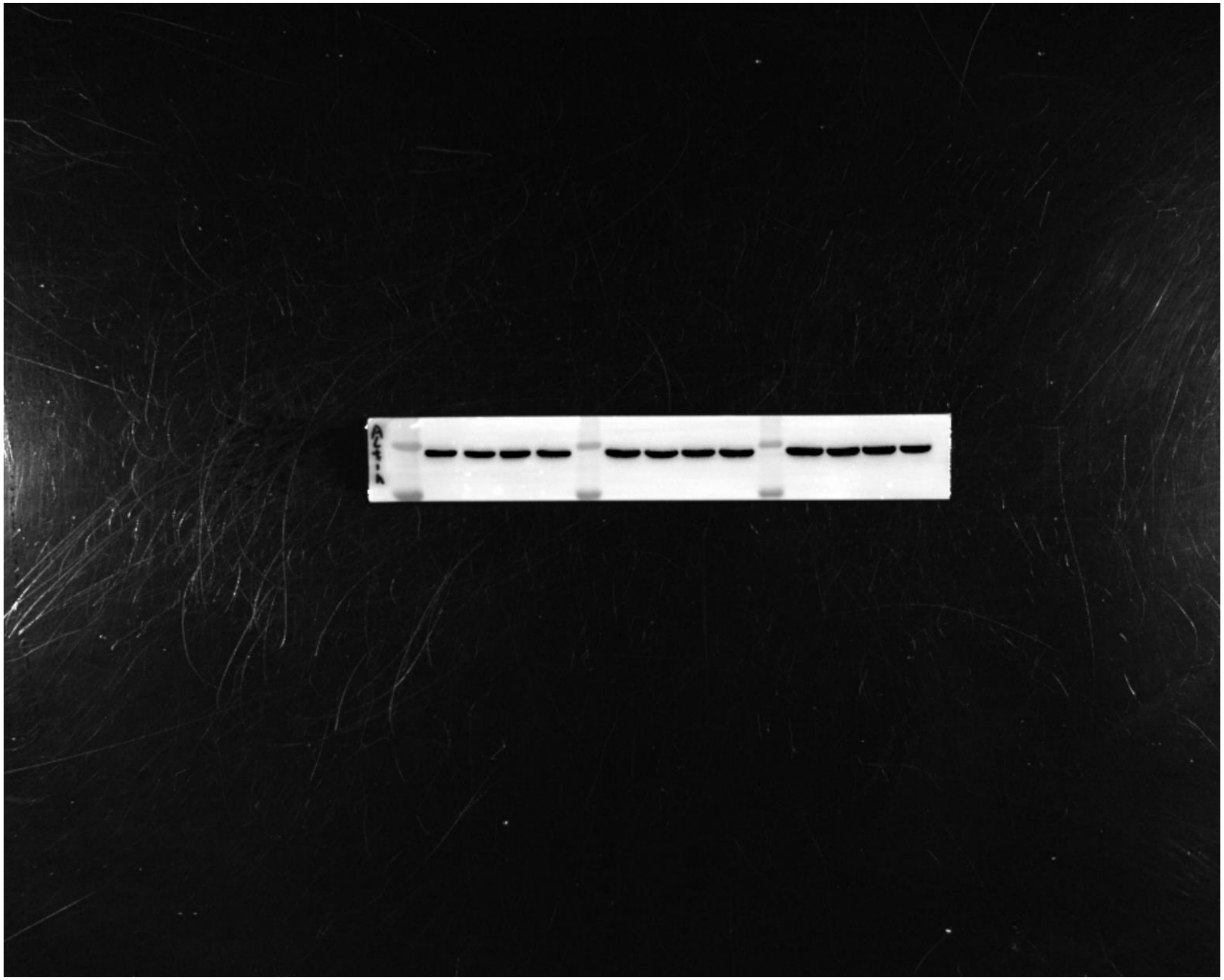

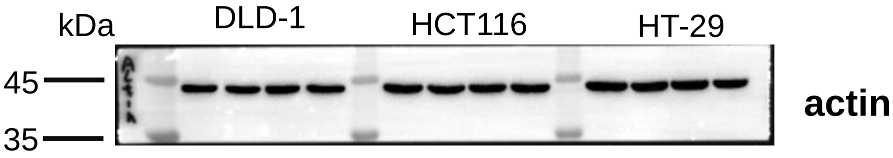

Fig. 5E

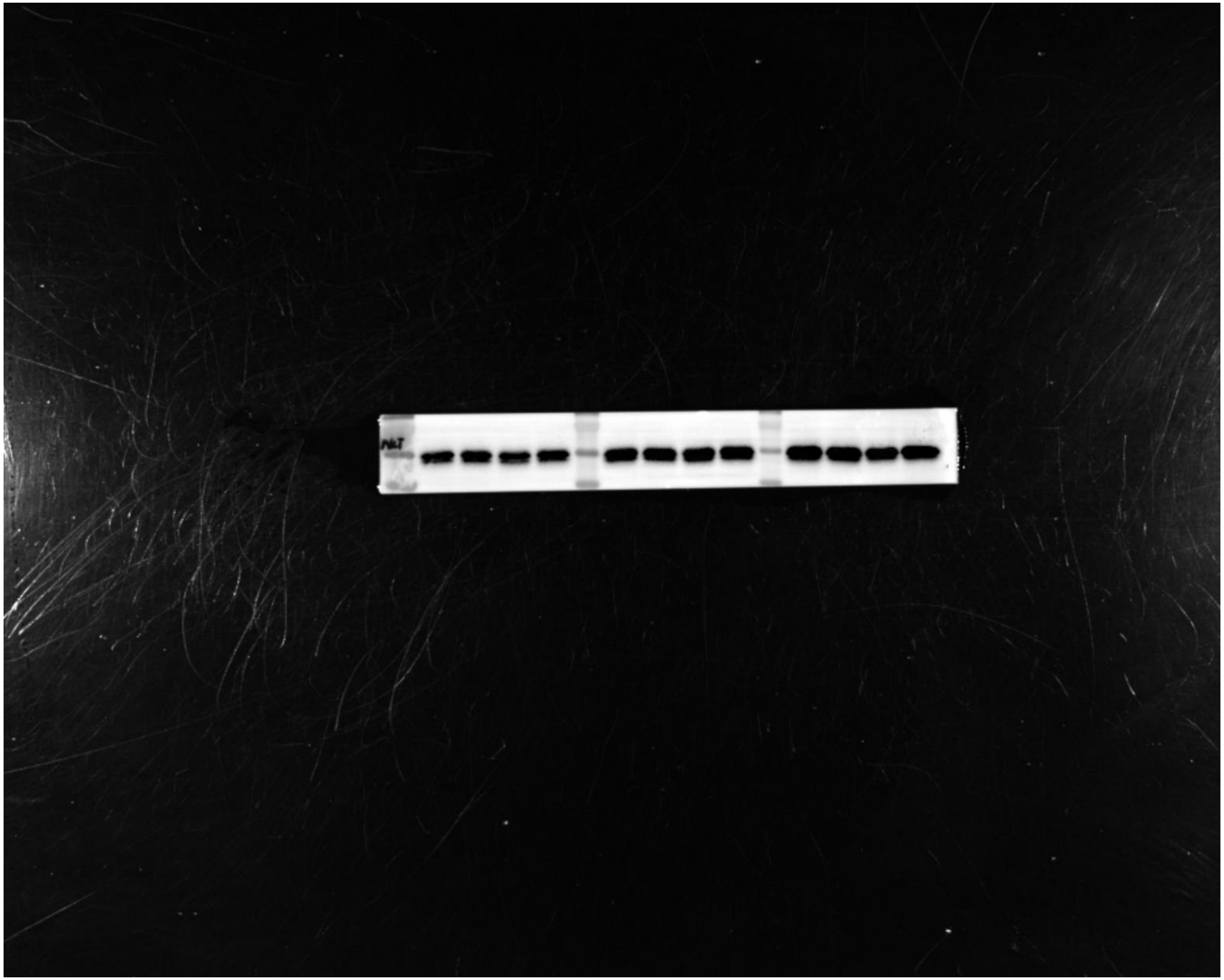

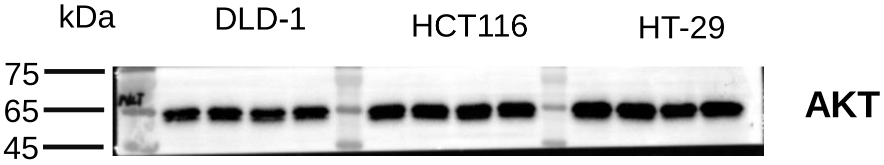

Fig. 6E

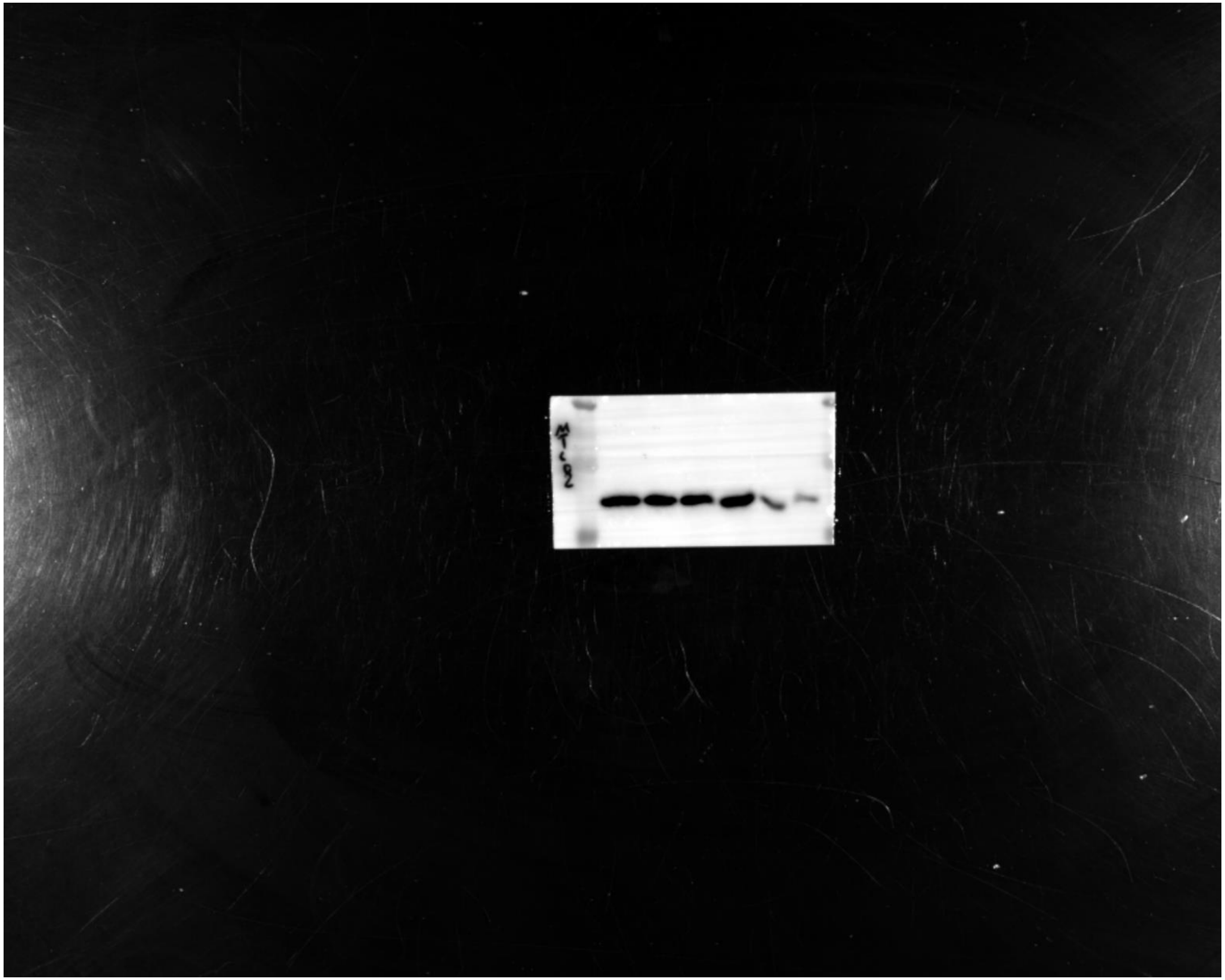

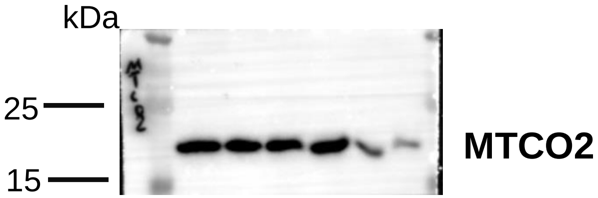

Fig. 6E

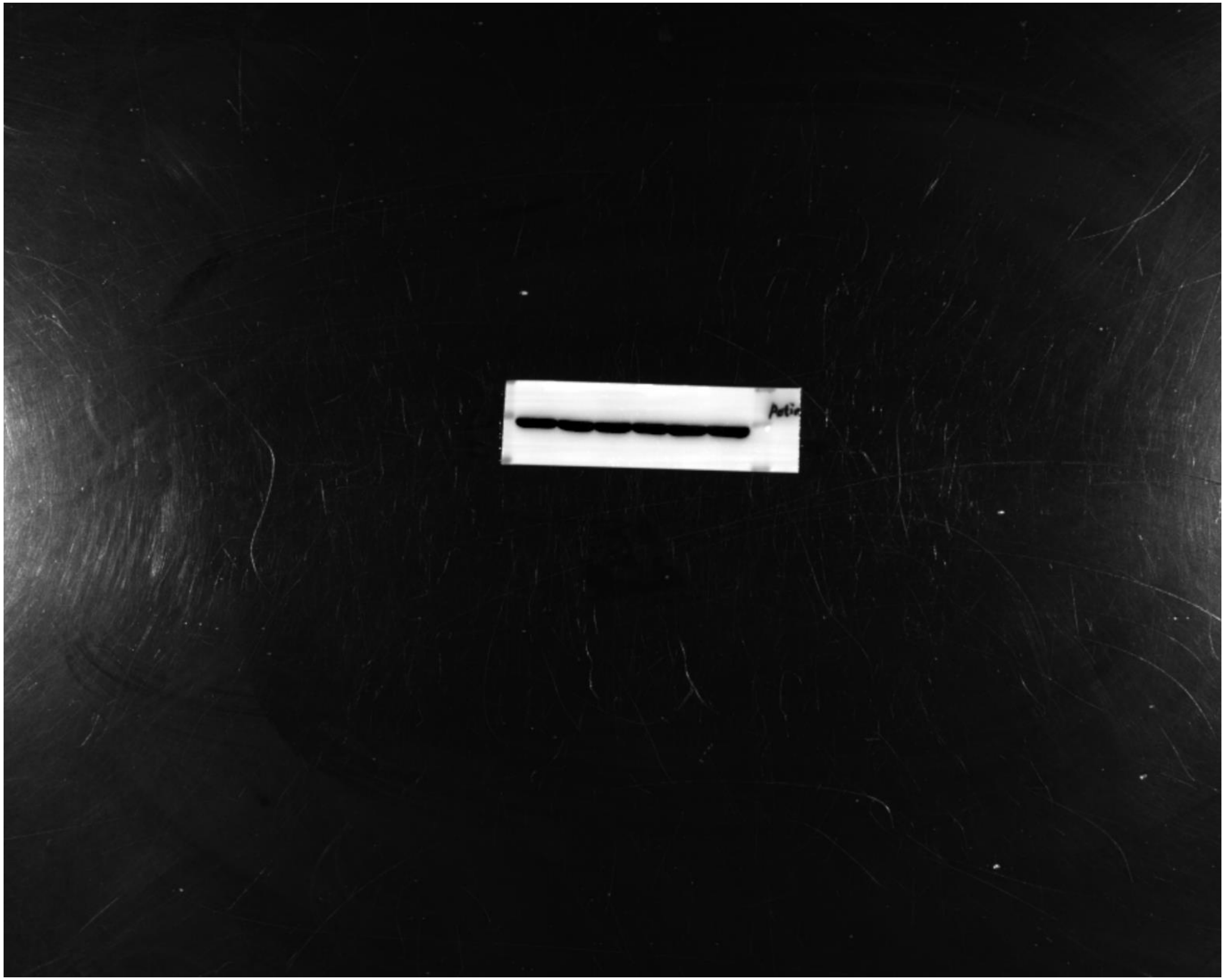

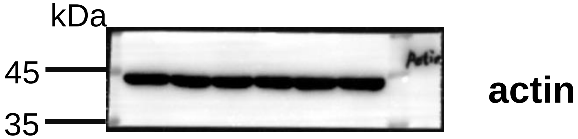

Figure S4A

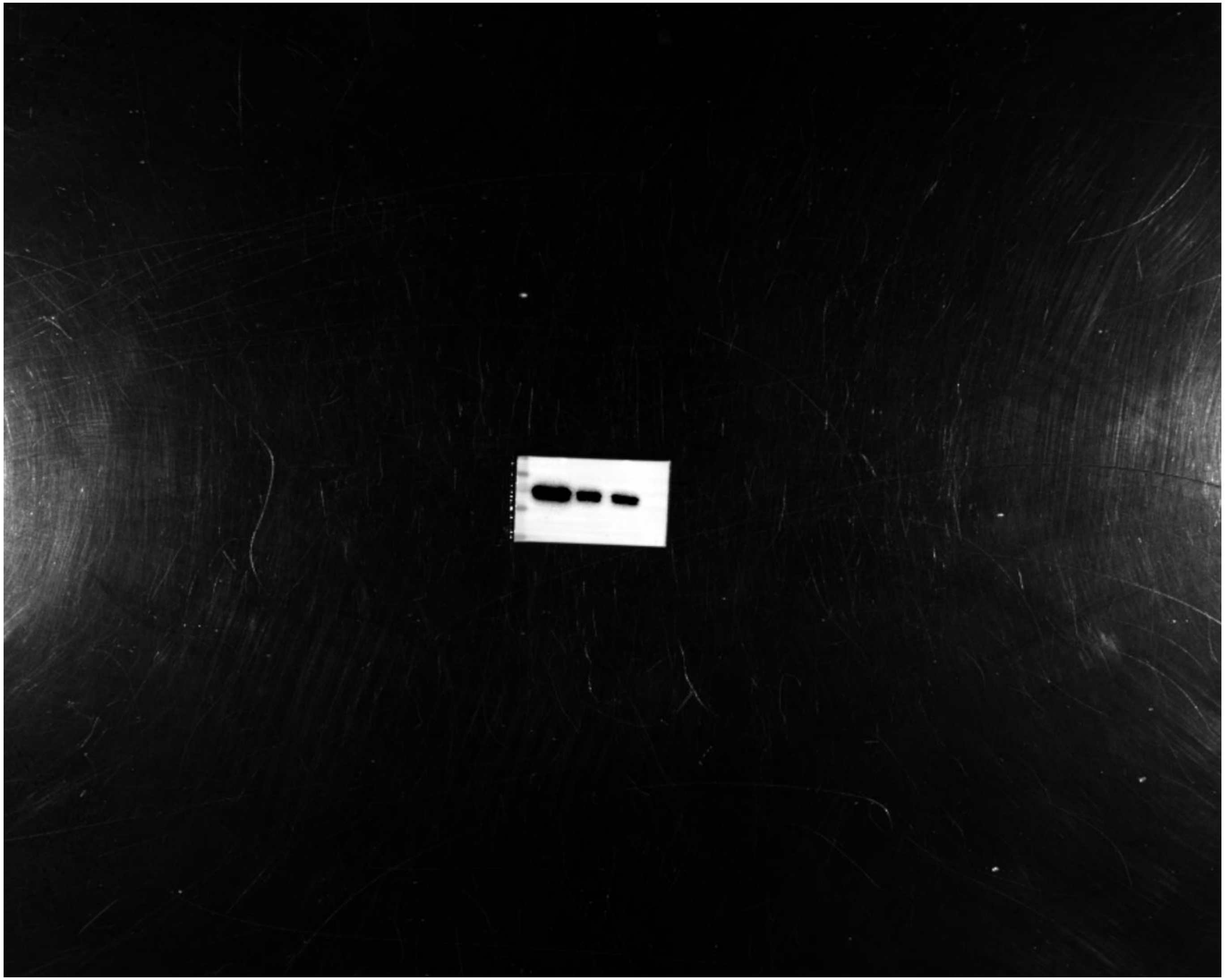

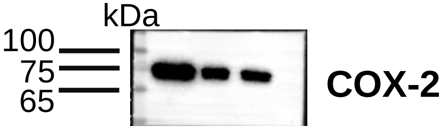

Figure S4A

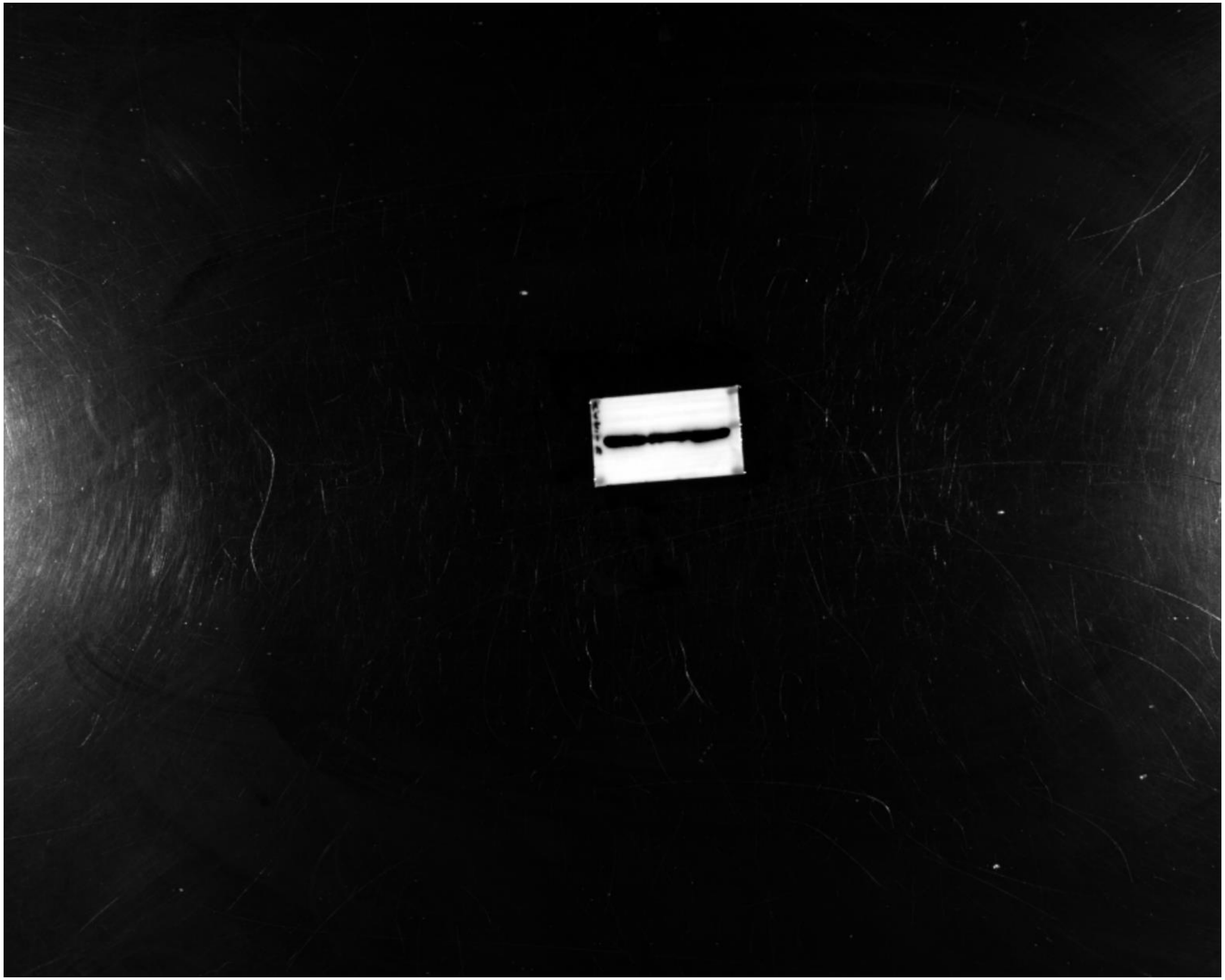

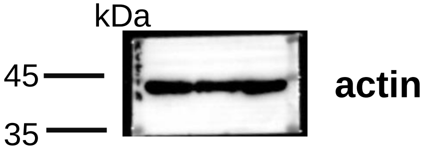

Figure S7

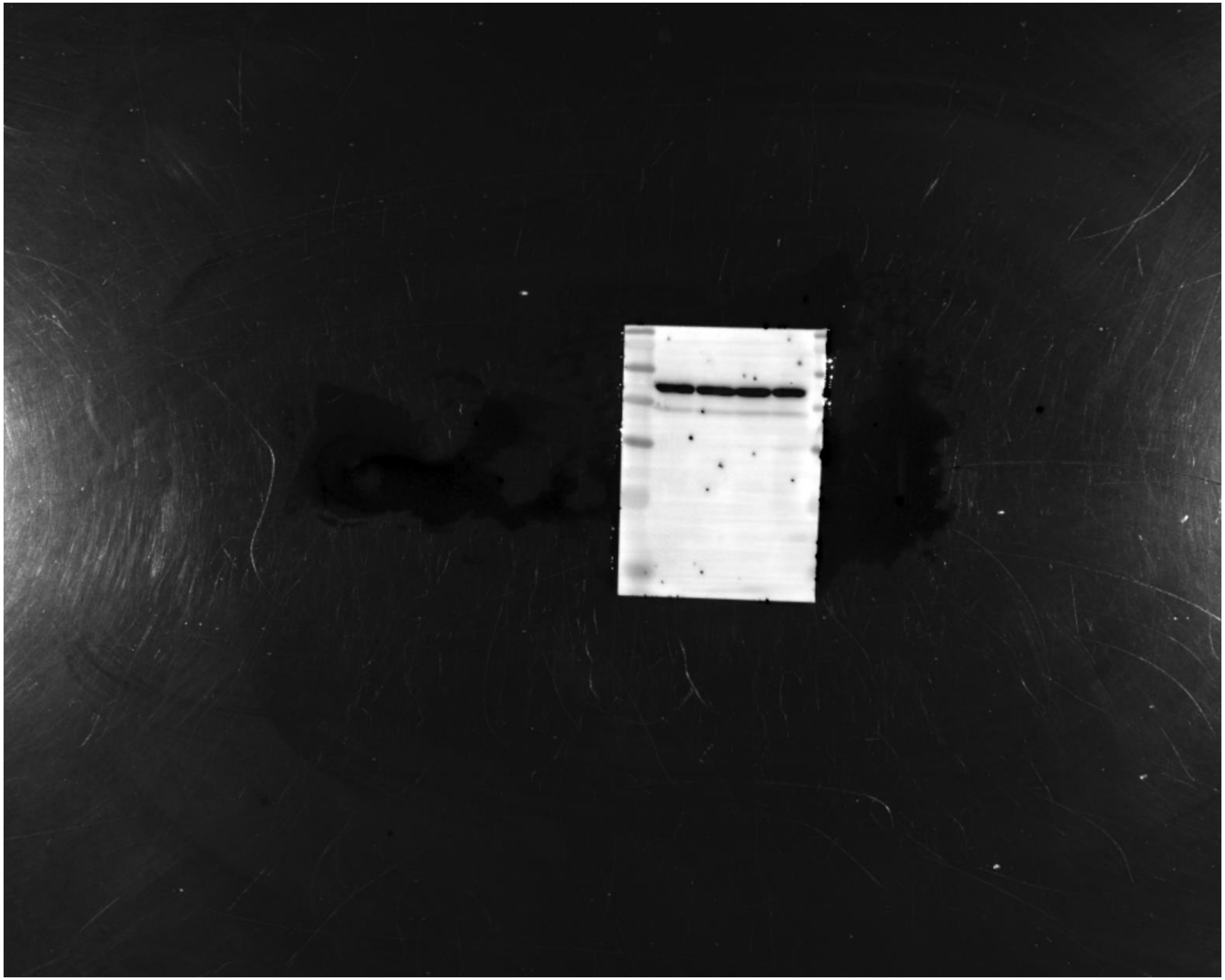

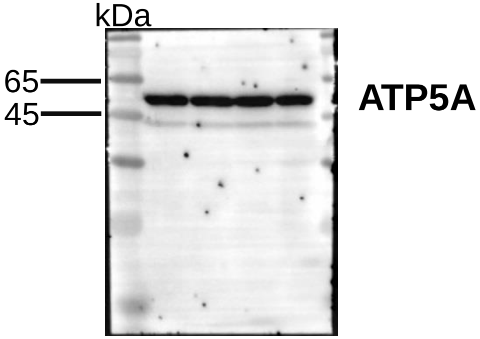

Figure S7

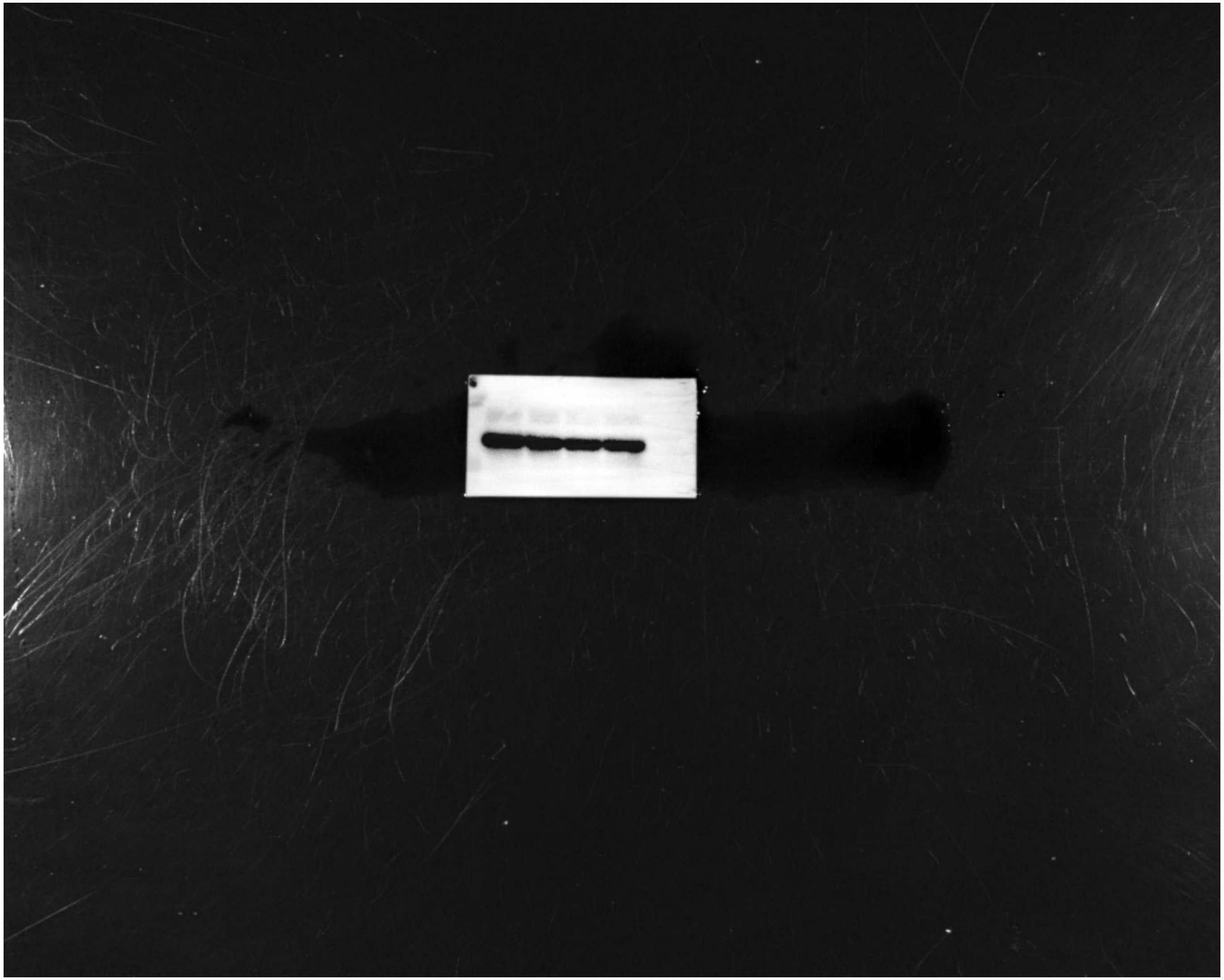

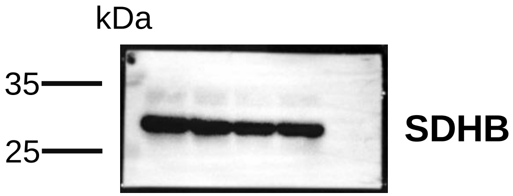

Figure S7

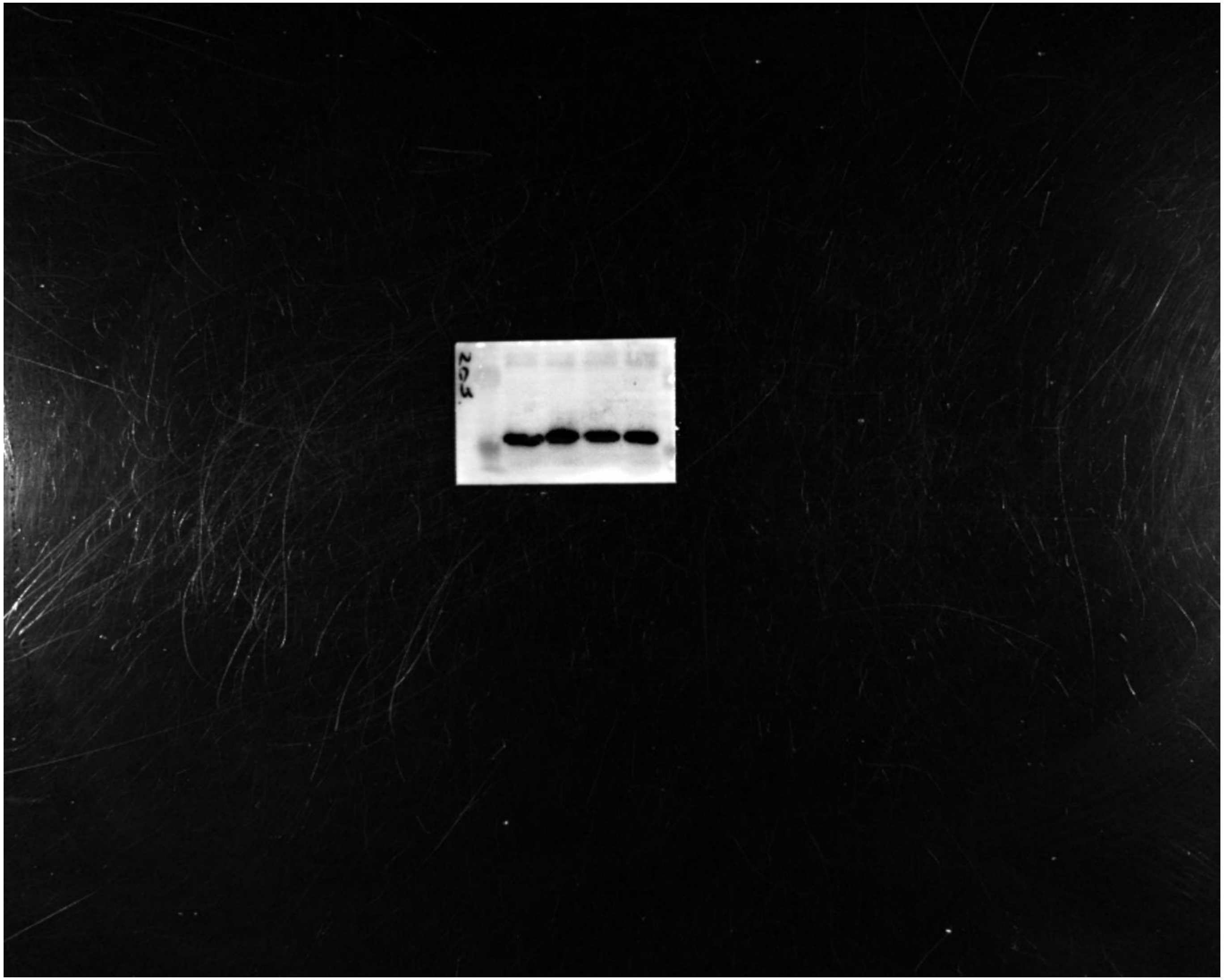

kDa

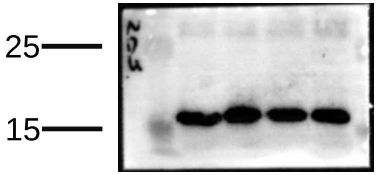

NDUFB8

Figure S7

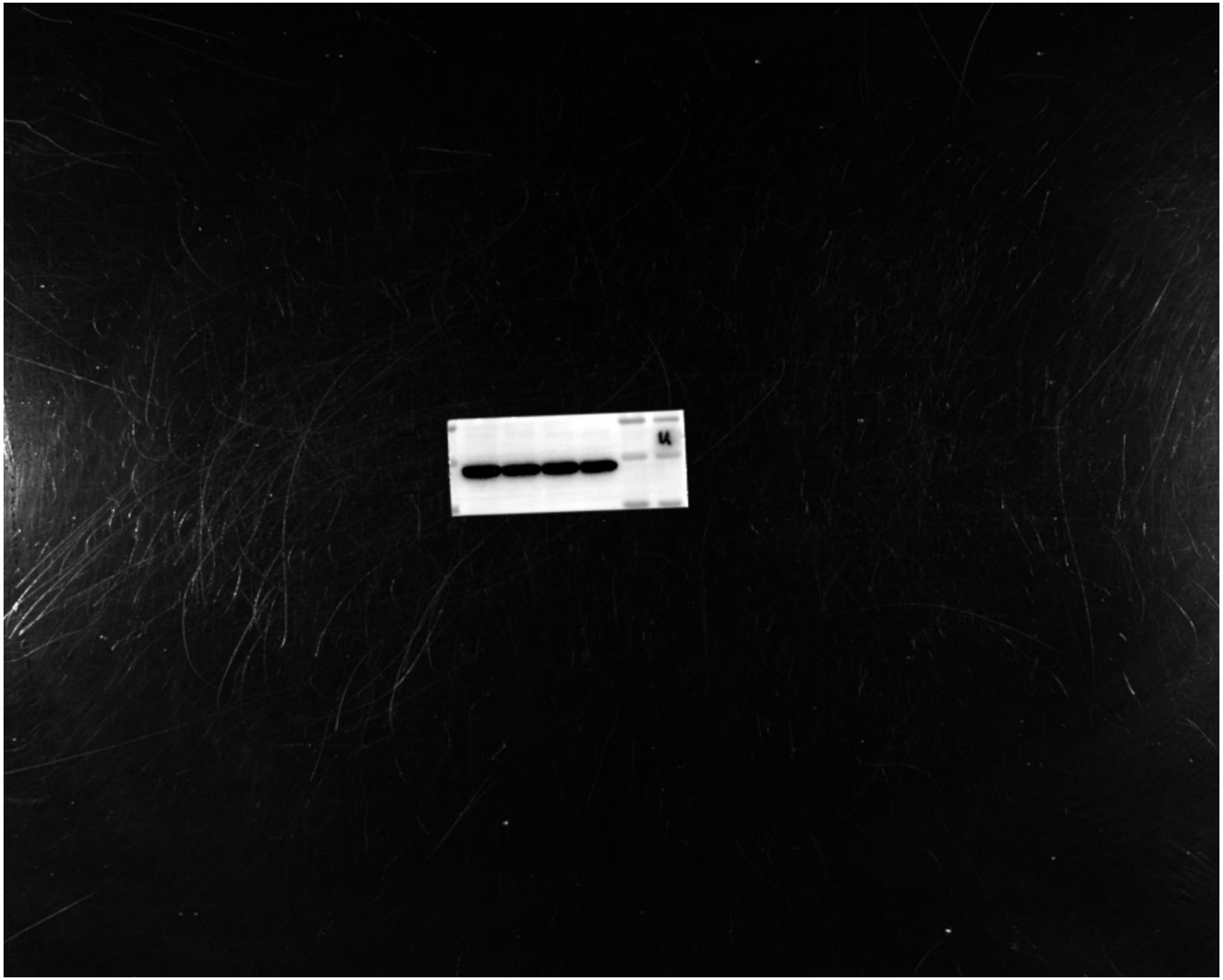

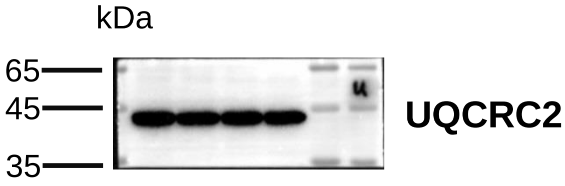

Figure S7

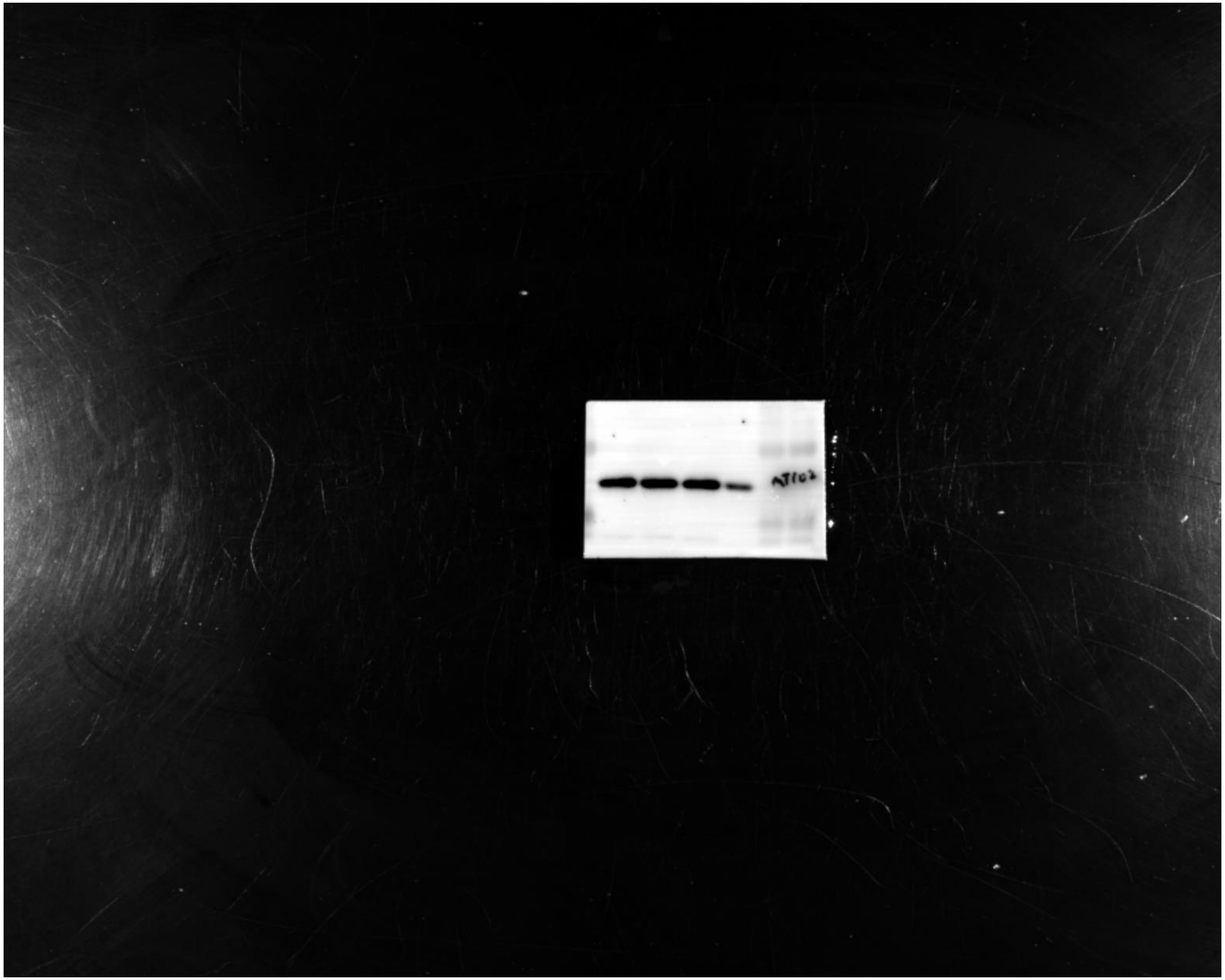

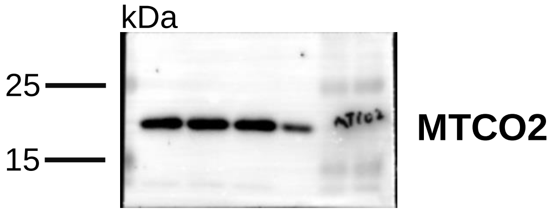

Figure S7

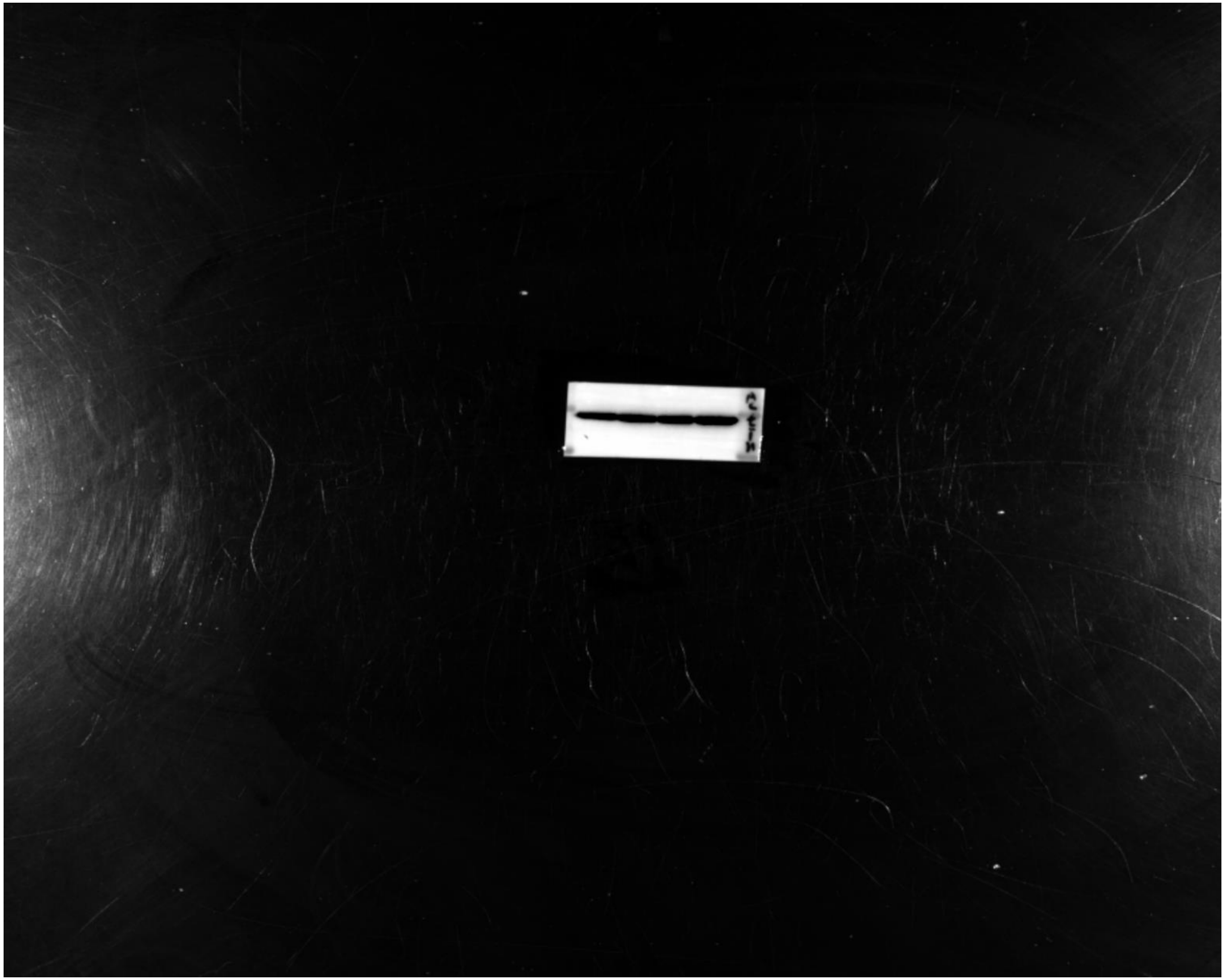

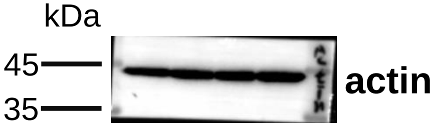

Supplement: Supplementary file 1 [file cancers-11-00931-s001.zip › Original Western Blot Pictures.pdf]
